# Multiple cytochrome P450 genes: conferring high levels of permethrin resistance in mosquitoes, *Culex quinquefasciatus*

**DOI:** 10.1038/s41598-021-88121-x

**Published:** 2021-04-27

**Authors:** Ting Yang, Ting Li, Xuechun Feng, Ming Li, Shikai Liu, Nannan Liu

**Affiliations:** 1 Department of Entomology and Plant Pathology 301 Funchess Hall, Auburn University, Auburn, AL 36849-5413 USA; 2 Department of Biology Sciences, University of California, San Diego, CA 92093 USA; 3 College of Fisheries, Ocean University of China, Qingdao, China

**Keywords:** Biochemistry, Biological techniques, Molecular biology, Physiology

## Abstract

Insecticides, especially pyrethroids, are the most important in the insect pest control and preventing insect vector-borne human diseases. However, insect pests, including mosquitoes, have developed resistance in the insecticides that used against them. Cytochrome P450s are associated with insecticide resistance through overexpression and detoxification mechanisms in insect species. In this study, we utilized a powerful tool, the RNAi technique, to determine the roles of key P450 genes overexpressed in permethrin resistant mosquitoes that confer insecticide resistance to unravel the molecular basis of resistance mechanisms in the mosquito *Culex quinquefasciatus.* The results showed that knockdown of 8 key P450 genes using RNAi techniques significantly decreased resistance to permethrin in resistant mosquitoes. In silico modeling and docking analysis further revealed the potential metabolic function of overexpressed P450 genes in the development of insecticide resistance in mosquitoes. These findings not only highlighted the functional importance of these P450 genes in insecticide resistance, but also revealed that overexpression of multiple P450 genes was responsible for the high levels of insecticide resistance in a mosquito population of *Culex quinquefasciatus*.

## Introduction

Mosquito-borne diseases are among the leading causes of human deaths worldwide^1^. Insecticides, especially pyrethroids, are the most important weapon in our arsenal for the control of mosquito vectors and mosquito-associated diseases, particularly those for which no vaccines have yet been developed^2,3^. Insecticides, especially pyrethroids, play a major role in campaigns to eradicate mosquito-borne diseases worldwide, however, the wide-spread development of resistance in mosquitoes to commonly used insecticides, in conjunction with the huge increase in human mobility seen in recent years, is leading to worldwide outbreaks of mosquito-related diseases^2,4^. Studies have revealed that mosquitoes can develop insecticide resistance through the transcriptional overexpression and/or enzymatic activity elevation of P450s^2,5^. P450s are critical for the detoxification and/or activation of xenobiotics, such as insecticides and plant toxins^6,7^. Overexpression of P450 genes can significantly affect the disposition of xenobiotics in the tissues of organisms, altering their pharmacological/toxicological effects^8^. New technologies such as whole-genome sequencing, RNA sequencing, deep targeted sequencing, and microarrays are allowing researchers to identify genes at a genome level that are potentially involved in insecticide resistance, through which multiple P450 genes are identified overexpressed mosquito species, including *Cx. quinquefasciatus*^9–12^, *Anopheles gambiae*^13^, and *Aedes aegypti*^14^.

To pinpoint the specific role of P450 genes in resistance, several biological technologies have been employed to investigate the gene functions, such as RNA interference (RNAi), a powerful and robust tool that discovers gene functions by using double-stranded RNA (dsRNA) to disrupt the target mRNA^15^, transcription activator-like effector nucleases (TALEN)s, and clustered regularly interspaced short palindromic repeats (CRISPR/Cas9)^16^. These techniques have made great contribution and applied successfully in many insect species to investigate the specific functions played by individual genes, including development^17^, metamorphosis^18^ and reproduction^19^, as well as to characterize the gene functions in the development of insecticide resistance in insect pests such as the mosquitoes *Cx. quinquefasciatus*^16,20,21^, *Anopheles gambiae*^22^ and *Aedes aegypti*^23^, the cockroach *Blattella germanica*^24^, the common bedbug *Cimex lectularius*^25^, and the aphid *Sitobion avenae*^26^.

Several P450 genes have been shown to be overexpressed in resistant *Cx. quinquefasciatus*^9^. In this study, we further characterized the function of these overexpressed P450 genes, revealing the association between changes in the expression levels of these P450 genes and the levels of insecticide resistance in mosquitoes using RNAi and in silico modeling and docking analyses. Our findings not only highlighted the functional importance of these P450 genes in insecticide resistance, but also revealed that the high levels of insecticide resistance in a single mosquito population were conferred by the increased expressed of multiple P450 genes their detoxification.

## Results

### Knockdown of the specific cytochrome P450 gene associated with larval resistance of *Cx. quinquefasciatus* to permethrin

**Figure 1:**
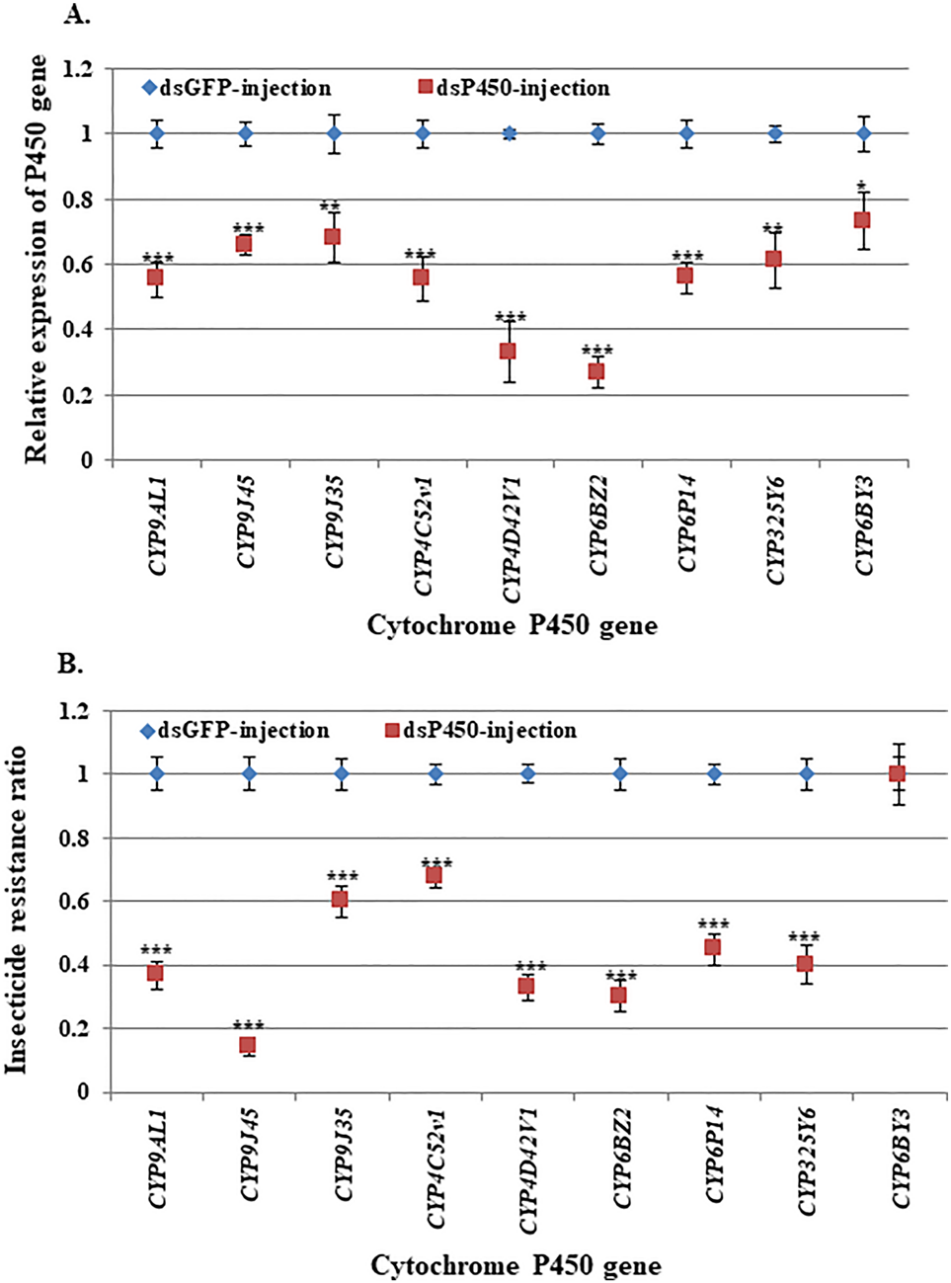
Knockdown of P450 genes associated with permethrin resistance in larvae of *Cx. quinquefasciatus*. RNAi was conducted in the larvae of the permethrin resistant mosquito strain, HAmCq^G8^. DsRNA of P450 and GFP genes were individually injected into 3^rd^ instar larvae of *Culex* mosquitoes. (**A**) Gene expression was detected by real-time PCR in 4^th^ instar larvae, showing significantly decreased expression of P450 genes compared with GFP injection mosquitoes. (**B**) Permethrin sensitivity was determined by larval bioassay, indicating increased susceptibility to permethrin in dsRNA of 9 P450 gene injection mosquitoes compared with GFP-injection ones; the negative control showed no increased susceptibility to permethrin in the dsRNA of non-overexpressed P450 gene injection mosquitoes. The data shown are the mean ± SEM (n ≥ 3). The Student’s *t*-test was used for significance analysis. *P < 0.05; **P < 0.01; ***P < 0.001.

The functional study of the role played by the up-regulated P450 genes in the insecticide resistance of *Culex* mosquitoes was conducted by using the RNAi technique to knock-down each of the specific P450 genes in the late 3^rd^ or early 4^th^ instar larvae of HAmCq^G8^. RNAi on the 8 P450 genes in the larvae of HAmCq^G8^ mosquitoes showed that the RNAi reduced the mRNA levels of *CYP9AL1* to 0.55-, *CYP9J45* to 0.65-, *CYP9J35* to 0.70-*, CYP4C52V1* to 0.55-*, CYP4D42V1* to 0.35-*, CYP6BZ2* to 0.30-*, CYP6P14* to 0.55-*, CYP325Y6* to 0.60- and CYP6BY3 (negative control) to 0.70-fold in comparison with the effect of dsRNA on GFP injection mosquitoes (Fig. 1a), suggesting the expression of these P450 genes has been successfully suppressed by RNAi. A parallel RNAi functional study on a non-overexpressed P450 gene, namely *CYP6BY3*, was conducted as a negative control. The results showed a significant decrease in the expression of *CYP6BY3*, dropping to 0.70-fold in dsRNA-*CYP6BY3* injected HAmCq^G8^ larvae.

To determine the relationship between the knock-down of these P450 genes and permethrin resistance in the HAmCq^G8^ strain, we performed a larval bioassay to test the resistance levels in dsRNA for the P450 gene- and GFP-injection HAmCq^G8^ larvae. As shown in Fig. 1b, the LC_50_ of the dsRNA-P450 injected mosquitoes was significantly decreased in comparison with the dsRNA-GFP injected mosquitoes, with resistance ratios decreasing to 0.37 for *CYP9AL1*, 0.18 for *CYP9J45*, 0.60 for *CYP9J35*, 0.65 for *CYP4C52V1*, 0.34 for *CYP4D42V1*, 0.30 for *CYP6BZ2*, 0.49 for *CYP6P14*, and 0.40 for *CYP325Y6*. The negative control dsRNA-*CYP6BY3* injected mosquitoes showed no change in their sensitivity to permethrin (Fig. 1b). These results demonstrate a strong association between the suppression of specific up-regulated P450 genes and increased susceptibility to permethrin in resistant *Culex* mosquitoes. The similar result has also been found in our previous study, in which RNAi reduced the mRNA level of CYP9M10 to 0.44-fold and resulted in the decreased level of resistance to 0.54-fold^20,21^. In contrast to the RNAi technique, which could only partially reduce the mRNA levels of genes and results in partial decreased in the levels of resistance, completely knockout gene function of *CYP9M10* copy by ALEN and CRISPR/Cas9 had showed an approximately 110-fold reduction in permethrin susceptibility in resistant *Culex* mosquitoes^16^.

### Knockdown of the specific cytochrome P450 gene associated with adult resistance of *Cx. quinquefasciatus* to permethrin

**Figure 2:**
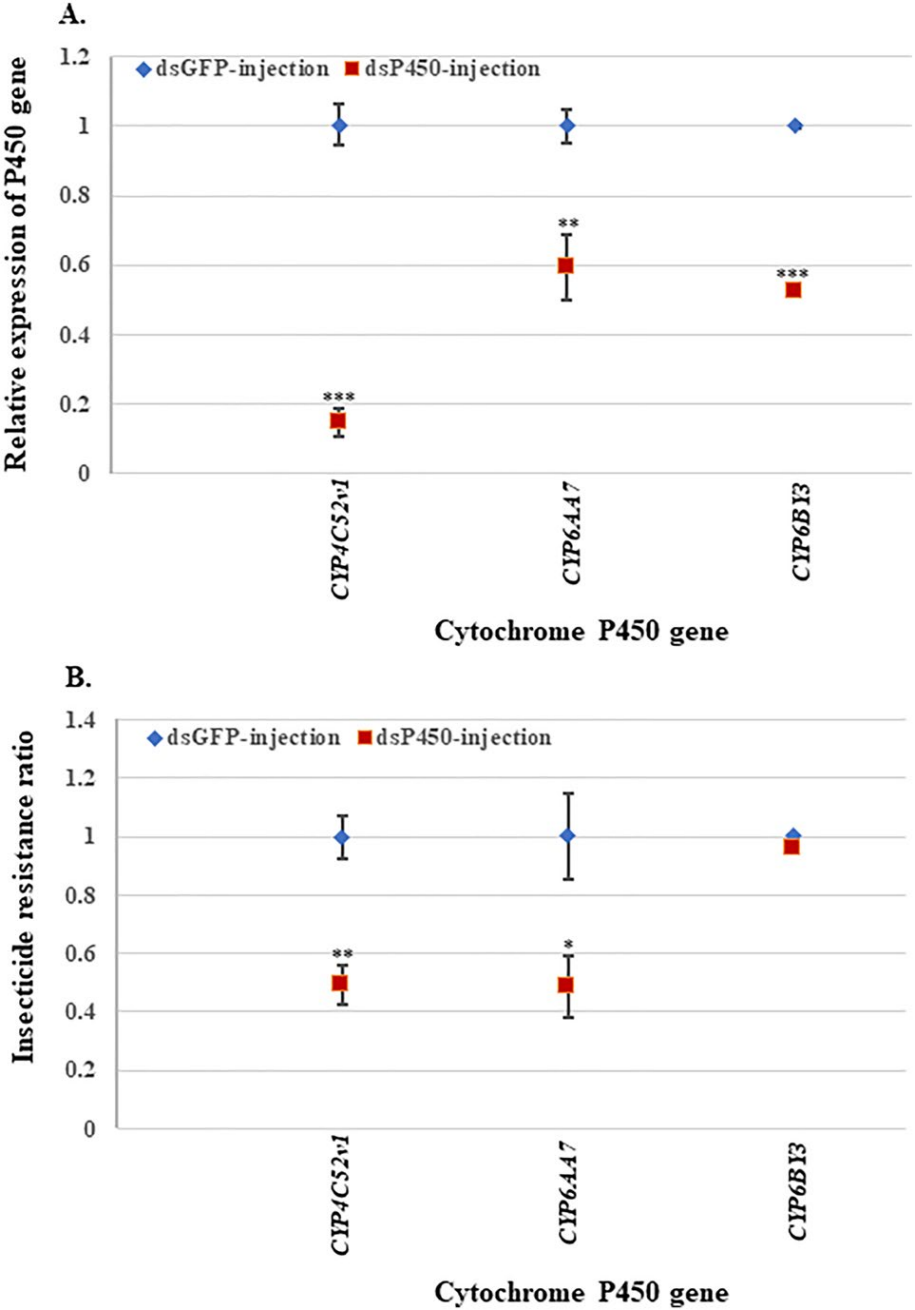
Knockdown of P450 genes associated with permethrin resistance in adults of *Cx. quinquefasciatus*. RNAi was conducted in the adults of the permethrin resistant mosquito strain, HAmCq^G8^. DsRNA of P450 and GFP genes were individually injected into 1-d-old female adults of *Culex* mosquitoes. (**A**) Gene expression was detected by real-time PCR in female adults, showing significantly decreased expression of P450 genes compared with GFP injection mosquitoes. (**B**) Permethrin sensitivity was determined by adult bioassay, indicating increased susceptibility to permethrin in dsRNA of 2 P450 gene injection mosquitoes compared with GFP-injection ones; the negative control showed no increased susceptibility to permethrin in the dsRNA of non-overexpressed P450 gene injection mosquitoes. The data shown are the mean ± SEM (n ≥ 3). The student’s t-test was used for significance analysis. *P < 0.05; **P < 0.01; ***P < 0.001.

Since two P450 genes, namely *CYP4C52v1* and *CYP6AA7*, were up-regulated in both the larval and adult stages of HAmCq^G8^, we then moved on to conduct RNAi in the female adults of the resistant mosquitoes. After injection of dsRNA-*CYP4C52v1,* -*CYP6AA7*, and the dsRNA-GFP control into adult mosquitoes, dynamic changes in the gene expression were detected and the adult resistance to permethrin assayed. The results again showed significantly reduced expression levels, with *CYP4C52v1* dropping to 0.15 and *CYP6AA7* to 0.59 (Fig. 2a). The suppression of the P450 gene by RNAi once again resulted in increased susceptibility to permethrin, with resistance ratios falling to 0.49 for both *CYP4C52v1* and *CYP6AA7* in comparison with the mosquitoes receiving a dsRNA-GFP injection (Fig. 2b). In the parallel RNAi study with *CYP6BY3*, the successful knockdown of *CYP6BY3* had no effect on the level of resistance to permethrin in HAmCq^G8^ female adults (Fig. 2).

### Homology modeling and substrate docking

**Figure 3:**
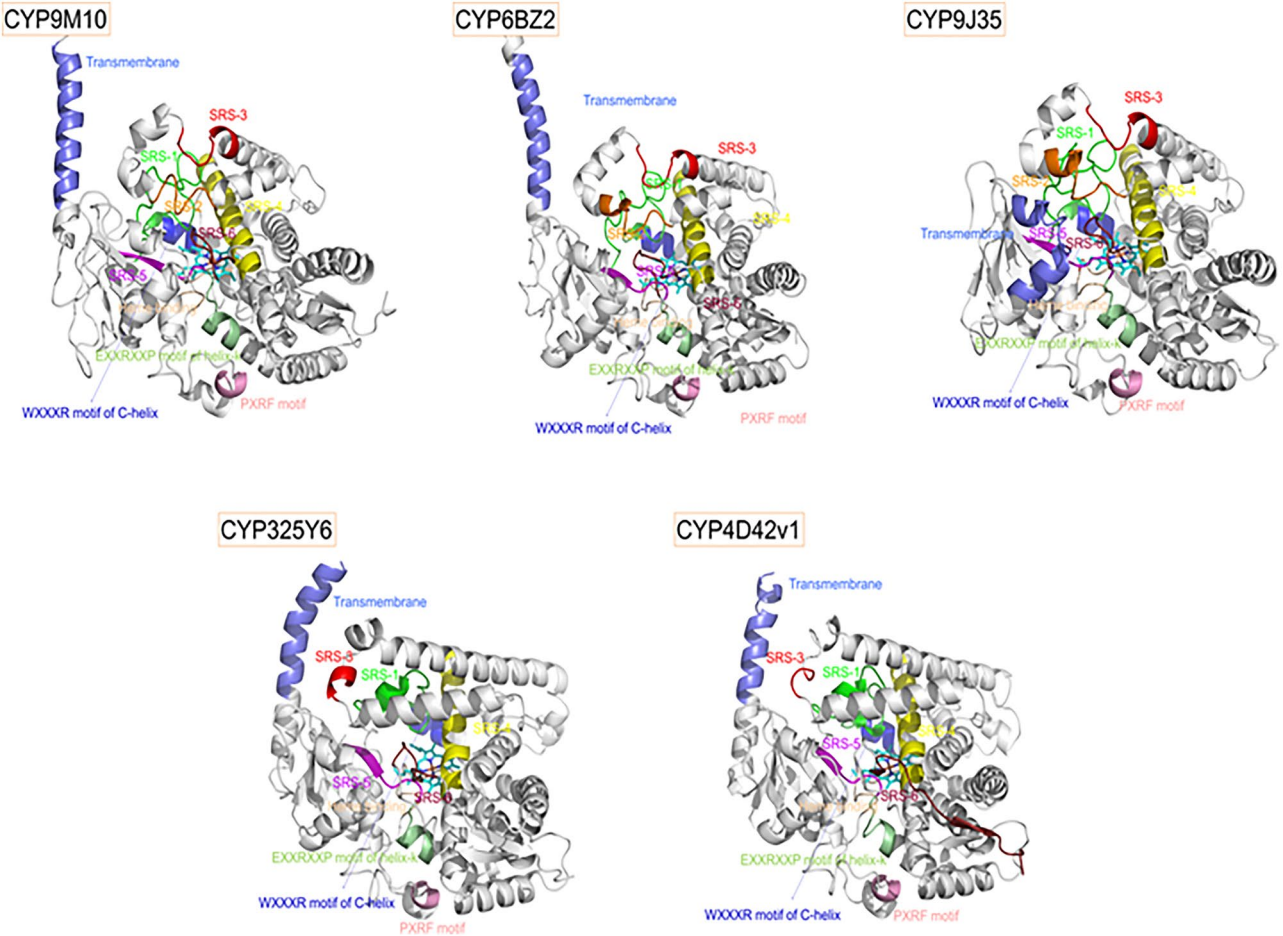
Homology modeling of CYP450s. The homology models for CYP9M10, CYP6BZ2, CYP9J35, CYP325Y6 and CYP4D42v1 were constructed with conserved SRSs. Motifs are labeled. Six putative SRSs are colored and predicted, according to Gotoh’s predicted models^43^. SRS1 to 6 are represented by green, orange, red, yellow, purple and ruby, respectively. The WXXXR motif of C-helix is shown in blue. The EXXRXXP motif of helix-k is shown in pale green. The PXRF motif is shown in pink and the Heme binding motif is shown in pale yellow. The HEME group is represented by cyan sticks.

**Figure 4:**
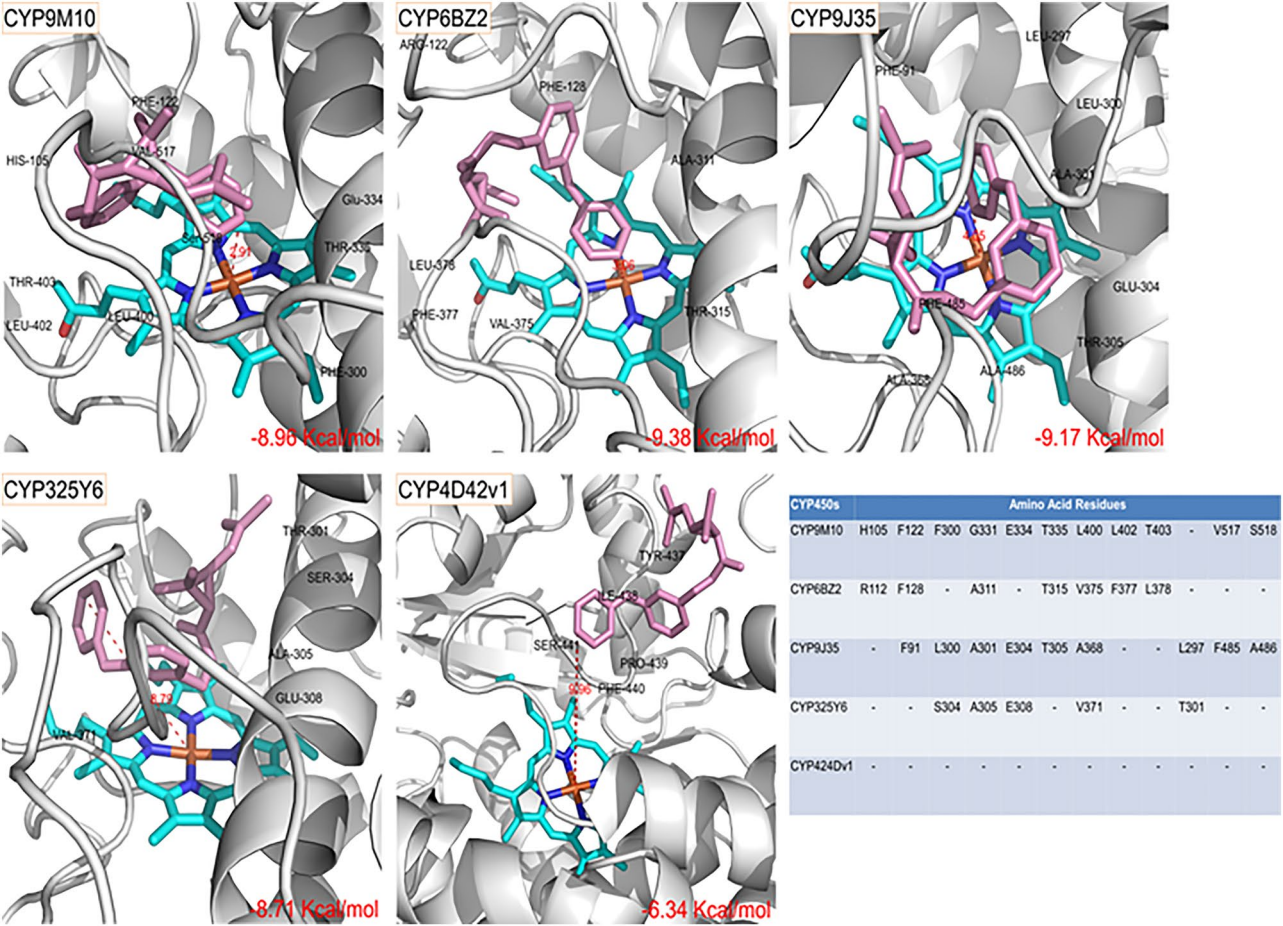
Docking models of permethrin in the active sites of CYP450s. The predicted binding models of permethrin in the active sites of CYP9M10, CYP6BZ2, CYP9J35, CYP325Y6, and CYP4D42v1. The partial homology model of CYP450 is represented by white helices and sheets; the Heme group is represented by cyan sticks, and the permethrin ligands are shown as pink sticks. The conserved amino acids of the catalytic pockets are labeled and summarized.

In a previous study, two P450 genes, *CYP9M10* and *CYP6AA7*, was found to perform a critical function in the insecticide resistance^21^ and metabolism of permethrin in mosquitoes^27^. We, therefore, utilized in silico 3D modeling and docking to further confirm the metabolic function of CYP9M10 in insecticide resistance. The homology modeling and ligand docking studies for CYP9M10, CYP6BZ2, CYP9J35, CYP325Y6, and CYP4D42v1 showed several conserved P450 characteristics, including substrate recognition sites 1–6 (SRS1–SRS6), the heme-binding structure, a WXXXR motif of the C-helix, and an EXXRXXP motif of the helix k, as well as a PXRF motif (Fig. 3). SRS-2 was not present in either CYP325Y6 or CYP4D42v1, and the SDS-3 was also different in these two models compared with that in the other three P450 models. Applying the Autodock v1.5.6 tools to investigate the binding of permethrin within these five CYP450 models, comparing the estimated binding affinity and distance between the 4′-hydroxylation site of permethrin and the heme iron center for each predicted binding mode (Fig. 4), with larger binding energies indicating lower binding affinities for ligands and proteins. The docking results revealed favorable binding affinities (− 8.96 kcal/mol for CYP9M10, − 9.38 kcal/mol for CYP6BZ2 and − 9.17 kcal/mol for CYP9J35) and distances (≤ 6.0 Å) between the heme iron centers and permethrin 4′-carbon hydroxylation sites (2.91 Å for CYP9M10, 3.06 Å for CYP6BZ2 and 4.05 Å for CYP9J35), suggesting the strong binding affinity of these CYP450s towards permethrin insecticides and further demonstrating their close association with insecticide resistance in mosquitoes. However, the lowest binding affinities (− 6.34 kcal/mol) and greatest distance (9.96 Å) between the 4′ carbon hydroxylation site and heme iron center were observed for CYP4D42v1 and a relatively long binding distance (8.79 Å) was also found for CYP325Y6, indicating the weak binding affinity of permethrin within the structures of these two P450s. Their involvement in permethrin resistance may thus be due to an increase in the gene expression level rather than a change in binding affinity. Residues in the catalytic pocket of these five CYP450s were also examined using LigPlus (http://www.ebi.ac.uk/thornton-srv/software/LigPlus/)^28^. The amino acid residues Gly331/Ala301/Ala305/Ala311 and Leu400/Ala368/Val371/Val375 found in CYP9M10, CYP9J35, CYP325Y6, and CYP6BZ2, respectively, are highly conserved in all four of these CYP450s; other amino acids, such as Phe91/122 and E334/304/308, are conserved in either two or three of the CYP450s (Fig. 4). The active sites of these four CYP450s are rich in hydrophobic amino acids such as Valine, Phenylalanine, Alanine, and Leucine, providing a favorable chemical environment for hydrophobic insecticide binding. The amino acid residues in the catalytic pocket of CYP4D42v1 are not conserved, which may be due to its low binding affinity as well as the greater distance between the permethrin ligand and the heme center of the protein structure.

## Discussion

Cytochrome P450s are known to be members of a superfamily of metabolic enzymes that is found in all living organisms^2^. The overexpression of P450 genes, resulting in increased levels of P450 proteins and P450 activities, has been associated with enhanced metabolic detoxification of insecticides and has been implicated in the development of insecticide resistance in insects, including mosquitos^2,4,6,29^. Zhu et al.^16^ observed that several P450 genes, namely *CYP4D4v2*, *CYP4G2*, *CYP6A38*, and *CYP6A36*, were up-regulated by induction or constitutive expression in a permethrin resistant housefly strain ALHF. Cytochrome P450 genes have been identified as playing a role in pyrethroid resistance in mosquito species through gene overexpression mechanisms, including *CYP6Z1*^30^ and *CYP6P3*^31^ in *An. gambiae*, *CYP9J32* in *Ae. aegypti*^32^, *CYP4H34, CYP6F1*, *CYP9M10* and *CYP6AA7* in *Cx. quinquefasciatus*^9,11,33–35^. More interestingly, overexpression of *CYP9M10* gene has been identified in different strains of *Cx. quinquefasciatus* in different geography locations in Japan, USA, and South Africa^9–12,36,37^, indicating that some insect resistance P450 genes are global distributed and spread^37^. However, it is unclear how many P450 genes are involved in developing P450-mediated resistance in an organism such as the mosquito. The use of biological technologies, such as RNAi, TALENs, and CRISPR/Cas9^15–26^, provides valuable tools for gene functional studies that has now been applied in many insect species. In this study, we employed RNAi and in silico modeling and docking analysis to determine the roles of overexpressed P450 genes known to overexpressed in insecticide-resistant mosquitoes^9^ to unravel the molecular basis of resistance mechanisms in the mosquito *Cx. quinquefasciatus*. Eight overexpressed P450 genes, *CYP9AL1, CYP9J35, CYP9J45, CYP4C52v1, CYP4D42V1, CYP6BZ2, CYP6P14,* and *CYP6325Y6*, were knocked down in 3rd/4th instar larvae of HAmCq^G8^ mosquitoes by RNAi, revealing that decreased expression of these P450 genes corresponded with the decreased level of insecticide resistance to permethrin. These results demonstrate that the decreased resistance to permethrin of the mosquito larvae is strongly associated with each of the target P450 genes knocked down by RNAi, thus indicating that multiple cytochrome P450 genes are likely involved in permethrin detoxification in *Culex* mosquitoes. While there is always a possibility of knockdown of off-target P450s that would alter the resistant level of the mosquitoes^38^, our further Baculovirus expression and HPLC based metabolism studies of these P450 suggest that these enzymes metabolize permethrin in *Culex* mosquitoes^27^ (some of the metabolism data by Gong et al. are not yet published), further confirming the involvement of these P450s in permethrin detoxification in mosquitoes.

Although previous studies showed *Culex* mosquitoes had a higher resistance level to permethrin in 4th instar larvae than in adults compared to susceptible mosquitoes^39,40^, we anticipated that overexpressed P450 genes in adult resistant mosquitoes may perform a similar function in permethrin resistance as that observed in the larvae. Two P450 genes, *CYP4C52v1* and *CYP6AA7*, known to be overexpressed in HAmCq^G8^ adult mosquitoes^9^ were successfully knocked down by dsRNA injection in adult mosquitoes, again demonstrating a strong association of overexpressed P450 genes and levels of insecticide resistance. Similar results have been also reported in pyrethroid-resistant mosquito species such as *An. gambiae* for *CYP6Z1* and *CYP6P3* genes^29,41^ and *Anopheles funestus* for *CYP6P9* genes^42^. In addition, in silico 3D modeling and docking analysis revealed that CYP9M10, CYP6BZ2, and CYP9J35 all exhibited high binding affinities to permethrin, indicating the high potential of these genes for metabolizing insecticide in mosquitoes. Although CYP325Y6 and CYP4D42v1 failed to show a high level of binding affinity to permethrin, suppression of the gene expression caused decreased resistance to insecticide in resistant mosquitoes. Taken together, our findings not only highlighted the functional importance of these P450 genes in insecticide resistance, but also revealed that the high levels of insecticide resistance in a single mosquito population were conferred by the accumulative detoxification mechanisms resulting from increased expression of multiple P450 genes.

## Materials and methods

### Mosquito strains

A mosquito strain, HAmCq^G8^, was used in the current study to character P450 gene function in insecticide resistance using RNAi. The HAmCq^G8^ is the 8th generation of permethrin-selected offspring of a field-collected parental strain, HAmCq^G0^ collected from Huntsville, Alabama^9,34,39^. After 8 generation-selection with permethrin, resistance level in HAmCq^G8^ was 2700-fold compared with those of a susceptible strain, S-Lab^39^. Permethrin selections on HAmCq^G8^ mosquitoes have been continually conducted for every 6 months and insecticide bioassays and gene expression measurements using CYP6AA7 and CYP9M10 as the target gene are performed after each permethrin selection to diagnose the levels of resistance and resistance P450 gene up-regulation in these resistant strains. While it had been showed that two major resistance mechanisms were involved in the permethrin resistance in HAmCq^G8^, namely P450 mediated detoxification^9,33^ and target site insensitivity (*kdr*)^43,44^, the current study has been focused on the P450 mediated resistance. All the mosquitoes were reared at 25 ± 2 °C under a photoperiod of 12:12 (L:D) h. Adult females were fed blood samples from horses for egg development (supplied by the Large Animal Teaching Hospital, College of Veterinary Medicine, Auburn University). To maintain the resistance level of HAmCq^G8^, resistance selection with permethrin in 4th instar larvae were applied every half year.

### RNA extraction and cDNA preparation

Total RNAs were extracted from 4th instar larvae and female adults using the acidic guanidine thiocyanate (GIT)–phenol–chloroform method^45^. DNA in the RNA samples was removed using a TURBO DNA-free kit (Ambion) following the manufacturer’s instructions. cDNA was synthesized using the DNA-free RNA as a template and SuperScript II reverse transcriptase (Invitrogen), again following the manufacturer’s instructions. The quantity of cDNA was measured using a spectrophotometer prior to qRT-PCR. Each experiment was repeated more than 3 times with independent RNA preparation.

### Quantitative real-time PCR (qRT-PCR)

Each qRT-PCR reaction (15 μl final volume) consisted of 1 × SYBR Green master mix (Roche), 1 μl of cDNA, and a P450 gene specific primer pair designed according to each of the P450 gene sequences (http://cquinquefasciatus.vectorbase.org/) at a final concentration of 3–5 μM. The primer pairs are listed in Table 1. All samples, including the ‘no-template’ negative control, were performed in triplicate. Relative expression levels for the P450 genes were calculated by the 2^−ΔΔCT^ method using SDS RQ software^46^ and ABI7500 Real-Time PCR system (Applied Biosystems). The reaction cycle consisted of a melting step of 50 °C for 2 min, then 95 °C for 10 min, followed by 40 cycles of 95 °C for 15 s and 60 °C for 1 min. The 18S ribosome RNA gene, an endogenous control, was used to normalize the expression of target genes^20,21,47^. Each experiment was repeated at least three times with independent biological samples. The statistical significance of the gene expression was calculated using a Student's *t* test for all 2-sample comparisons and one-way analysis of variance (ANOVA) for multiple sample comparisons (SAS v9.1 software); a value of *P* ≤ 0.05 was considered statistically significant.

**Table 1:** Oligo nucleotides for qRT-PCR, dsRNA synthesis.

Transcript ID^a^	Accession no.	Gene^b^			Primer (5′–3′)^c^
CPIJ010540	XM_001855175.1	CYP9J35	qRT-PCR	Forward	TTGCCCAATGCTTCCTGTTCTTCC
				Reverse	GTTTCCGCCAAGTGCTTGTTCTGT
			dsRNA	Forward	TAATACGACTCACTATAGGGGAATCGACTCTACGCGGAAATA
				Reverse	TAATACGACTCACTATAGGGTTGACCTCCATAAGGGCTAAAC
CPIJ005955	XM_001847351.1	CYP6P14	qRT-PCR	Forward	AAGGTGGAACCAGGTCTGACGATT
				Reverse	CCATCATTAGCCGCGATTGCCTTT
			dsRNA	Forward	TAATACGACTCACTATAGGGCAACTCGCTTAAACAGCGCAACCT
				Reverse	TAATACGACTCACTATAGGGTTGGCTAGCTGAAACGGTGTTTGC
CPIJ012470	XM_001862711.1	CYP9AL1	qRT-PCR	Forward	TGAACGTCCTTAGGGATGGCGAAA
				Reverse	TTGCTAGTCGCGGAAACGAACTGA
			dsRNA	Forward	TAATACGACTCACTATAGGGACCCACGACGACTTCGTATC
				Reverse	TAATACGACTCACTATAGGGTCGATTACCCAAGCATAGCC
CPIJ005956	XM_001847352.1	CYP6BZ2	qRT-PCR	Forward	ACCATGGCGTCAGCTAAGGATGAA
				Reverse	TCCGTGGTGAATCCGACTAGCAAA
			dsRNA	Forward	TAATACGACTCACTATAGGGGGAAGGTTCCGCTGAAGTAAT
				Reverse	TAATACGACTCACTATAGGGCTTGTCCTGTATGTCCTGGTTC
CPIJ010537	XM_001855163.1	CYP9J45	qRT-PCR	Forward	ACCCACAGTACTTCCCAGA
				Reverse	CCGACAGCACCAACCTAAA
			dsRNA	Forward	TAATACGACTCACTATAGGGGCGGGACTACAACAACGAAT
				Reverse	TAATACGACTCACTATAGGGCATGTTGGGGGACCACTTAC
CPIJ007091	XM_001849018.1	CYP325Y6	qRT-PCR	Forward	AGCTACTGCTGGACGGAGTTCAAA
				Reverse	TCCTCAACCCAGCACTAAACGGAA
			dsRNA	Forward	TAATACGACTCACTATAGGGGCAACGACTTCACGGATAAGA
				Reverse	TAATACGACTCACTATAGGGCCCAGCACTAAACGGAATGT
CPIJ005959	XM_001847355.1	CYP6AA7	qRT-PCR	Forward	AAGGTTCGGGTTGAAGTCAGCTCT
				Reverse	TTGCGAGCTACGAAAGGAACTGGT
			dsRNA	Forward	TAATACGACTCACTATAGGGTACGAAGAACGGGAAGAAGCCGTT
				Reverse	TAATACGACTCACTATAGGGCCGGATTGTTGGCCAGTTCAAACA
CPIJ018943	XM_001869165.1	CYP4C52v1	qRT-PCR	Forward	AATGCCTCCAAGTTCTGCACTCCT
				Reverse	ATCGTTGTTGTTGTTGAGGTCGGC
			dsRNA	Forward	TAATACGACTCACTATAGGGCGGCAAAGATCGCAAATCTGCTCA
				Reverse	TAATACGACTCACTATAGGGTTCCAAGCTGTTCCTCCAGCTTCT
CPIJ009478	XM_001851374.1	CYP4D42V1	qRT-PCR	Forward	TCAACTATCTGGTTCGGGATGCGA
				Reverse	ACTTCCGGCTGAGGTTCGTTATGA
			dsRNA	Forward	TAATACGACTCACTATAGGGGGACACCTGGTCCTTCATAAC
				Reverse	TAATACGACTCACTATAGGGCCAACGTCACAAAGGGATAGA
CPIJ003375	XM_001844768.1	CYP6BY3	qRT-PCR	Forward	TCCACAAACTTGTAGCCGACACGA
				Reverse	GCAGCAGTCTCATCCAGCGTAAGA
			dsRNA	Forward	TAATACGACTCACTATAGGGCCGACACGATCGCGTACAG
				Reverse	TAATACGACTCACTATAGGGTCGCAAGTCCGACTCTGGA
	AY988447	18SRibosomal RNA	qRT-PCR	Forward	CGCGGTAATTCCAGCTCCACTA
				Reverse	GCATCAAGCGCCACCATATAGG

^a^The transcript ID number from the vectorbase of the *Cx. quinquefasciatus* genome sequence (http://cquinquefasciatus.vectorbase.org/).

^b^The annotation of the *Culex* P450 genes from http://drnelson.utmem.edu/CytochromeP450.html.

^c^Specific primer pair designed according to each of the P450 gene sequences of the *Cx. quinquefasciatus* in vectorbase (http://cquinquefasciatus.vectorbase.org).

### Double-stranded RNA synthesis

Several P450 genes are up-regulated in resistant strains of *Culex* mosquitoes; eight P450 genes, namely *CYP9AL1*, *CYP9J45*, *CYP9J35, CYP4C52V1, CYP4D42V1, CYP6BZ2, CYP6P14,* and *CYP325Y6* are up-regulated specifically in the larval stage of the permethrin-resistant HAmCq^G8^ strain in comparison with the lab susceptible strain (S-Lab) and field-collected parental strain (HAmCq^G0^), while two P450 genes, namely *CYP4C52v1* and *CYP6AA7*, are up-regulated in both the larval and adult stages in HAmCq^G8^ mosquitoes^9^. For the functional study of these up-regulated P450 genes in insecticide-resistant mosquitoes, we utilized the RNAi technique to suppress the expression of these specific P450s in order to further test their involvement in the development of insecticide resistance in *Culex* mosquitoes. To avoid a potential false-positive result due to the dsRNA gene injection effect, the dsRNA of a green fluorescent protein (GFP-pMW1650) gene was injected to serve as the control^20,21,47^, non-overexpressed P450-CYP6BY3 served as the negative control. Briefly, a 300–500 bp PCR product was generated that was complementary to the cDNA sequences of the specific P450 gene or pMW1650 plasmid, with T7 promoter sequences (5′-TAATACGACTCACTATAGGG-3′) adjacent to the 5′ ends of both the sense and antisense of each PCR primer (Table 1). The dsRNAs of the specific P450 genes and the GFP gene were synthesized by in vitro transcription using a MEGAscrip T7 High Yield Transcription kit (AB, Applied Biosystems) following the manufacturer’s instructions. Phenol/chloroform extraction followed by ethanol precipitation was used for the dsRNA purification method^45,46^. Each of the dsRNA-P450s was diluted in nuclease-free water to 3–4 μg/μL for injection.

### RNA interference conducted with dsRNA of specific P450 gene or GFP gene injection in HAmCq^G8^ larvae and adults

The 3rd instar larvae of HAmCq^G8^ were anesthetized in ice-cold water for about 5 min prior to injection. Anesthetized larvae were then placed on dry filter paper and 138 nl dsRNA (~ 400 ng) of the specific P450 or GFP gene was injected vertically to the body axis in the thoracic region using a capillary needle and Nanoject II (Drummond Scientific Company) under a dissection microscope. The capillary needle was pulled out using a needle puller (Sutter Instrument) following the program: Heat 545, Pull 30, Vel 120, Time 125. After injection, the larvae were immediately removed from the filter paper and placed back into distilled water under normal insectary rearing conditions and fed with yeast. For the mosquito adult microinjections, approximately 138 nl of dsRNA (~ 400 ng) was injected into the thorax of CO_2_-anesthetized 1-day-old female mosquitoes using a capillary needle, Nanoject II injector and Drosophila CO_2_ Fly Pads (Tritech Research, Inc.). Three day-post-injection, the living mosquitoes were collected and separated into two groups, the first of which was tested for dynamic changes of P450 gene expression using qRT-PCR detection system and the other was assayed for permethrin resistance using larva or adult bioassay. Each experiment was repeated > 3 times with independent microinjection and RNA isolation from other mosquitoes.

### Mosquito larvae and adult bioassays

Stock and serial dilutions of permethrin (94.34%, supplied by FMC Corp., Princeton, NJ) for the insecticide bioassay were prepared in acetone. The bioassay methods used for the larvae and adults were as described in previous studies^40,48^. Briefly, each bioassay consisted of twenty 4th instar mosquito larvae or 15 adults using 4–5 concentrations that resulted in > 0 and < 100% mortality. Control groups received only 1% acetone. Mortality was assessed after 24 h. At least 3 replications of the bioassay were performed. Bioassay data were pooled and analyzed by standard probit analysis, as described by Liu et al.^47^. The resistance ratio was calculated based on the LC_50_ of dsRNA-P450-injected mosquitoes divided by the LC_50_ of GFP -injection mosquitoes.

### In silico modeling and docking analysis

In silico 3D modeling was constructed by the I-TASSER server^49,50^ and five models were predicted for each P450. The top-scoring model for each P450 was then submitted to the FG-MD server for fragment guided molecular dynamics structure refinement^51^. Ligand permethrin structures were downloaded from the ZINC database (http://zinc.docking.org/) and prepared for docking using Autodock Tools v1.5.6 (http://mgltools.scripps.edu/downloads). Molecular docking was performed by Autodock 4.2^52^. For all dockings, a search space with a grid box of 60 × 60 × 60A centered at the heme iron center was set corresponding to substrate recognition sites (SRSs) following those of the CYP2 family proposed by Gotoh^53^. The figures were generated by Pymol for publication (http://www.pymol.org)^54^.
